# A survey on the anxiety status of adolescents in minority concentration areas of Western Hunan—three years after the onset of the COVID-19 pandemic

**DOI:** 10.3389/fpubh.2025.1509339

**Published:** 2025-04-25

**Authors:** Shengji Liu, Jiayi Zhang, Hongwei Chen, Lijun Chen

**Affiliations:** ^1^School of Psychology, Shandong Normal University, Jinan, China; ^2^Department of Culture and Arts, Lunan Technician College, Linyi, China

**Keywords:** COVID-19, anxiety, minority adolescents, self-efficacy, Western Hunan

## Abstract

The COVID-19 pandemic has had a profound impact on adolescents of all ethnic groups in China, with most studies focusing on Han Chinese adolescents. This study assesses the anxiety status of 426 high school students aged 15–18 from the Tujia and Miao Autonomous Prefecture of Western Hunan, a minority concentration area, 3 years after the pandemic began, and explores its influencing factors. Using a cross-sectional survey design, data were collected via questionnaires covering anxiety, parental migration, parental education level, and individual general self-efficacy. Descriptive statistics, t-tests, correlation analysis, and linear regression were used for data analysis. The results revealed that: (1) the prevalence rate of anxiety symptoms among the surveyed subjects was 60.4%, with moderate anxiety accounting for 16.4% and severe anxiety for 5.3%; (2) female students scored significantly higher in anxiety than male students; (3) left-behind children exhibited significantly higher levels of anxiety symptoms compared to non-left-behind children; and (4) correlation and regression analyses indicated that gender, being a left-behind child, and general self-efficacy can effectively predict anxiety status. These findings demonstrate that 3 years after the outbreak of the pandemic, the anxiety status of high school students in the minority concentration areas of Western Hunan remains relatively severe, with females, left-behind children, and individuals with low self-efficacy being at higher risk. The study provides a deeper understanding of the psychological conditions of minority adolescents in Western Hunan and offers scientific evidence for the development of targeted mental health intervention measures.

## 1 Introduction

Since its outbreak at the end of 2019, the COVID-19 pandemic has had far-reaching impacts on global society. This large-scale pandemic has not only posed significant challenges to public health systems but also brought major disruptions to the global economy and social life. Governments worldwide have implemented strict containment measures, such as home quarantine and school closures, to slow the spread of the virus. While these measures have been effective in controlling the pandemic, they have also had adverse effects on mental health (1). Adolescents, in particular, have faced multiple stressors during the pandemic, including academic pressure, social restrictions, and changes in family environments, leading to increasingly prominent mental health issues, with anxiety symptoms being especially prevalent (2, 3). According to a 2022 World Health Organization (WHO) scientific brief, the global prevalence of anxiety and depression increased by 25% during the first year of the COVID-19 pandemic. Similarly, studies in China have found that the outbreak of COVID-19 has led to an increase in anxiety levels among the Chinese public (4).

China is a multi-ethnic country, and while some studies have reported that the levels of anxiety and depression among Chinese adolescents have decreased and shown positive trends in the later stages of the pandemic (5), the anxiety status of adolescents in minority concentration areas remains underreported-a knowledge gap with significant implications for health equity. Our study addresses this disparity by focusing on anxiety levels among high school students in Western Hunan’s Tujia and Miao Autonomous Prefecture, a mountainous region marked by economic underdevelopment, linguistic diversity, and one of China’s highest rates of left-behind adolescents due to labor-driven parental migration. This region’s unique vulnerabilities create a synergistic matrix for prolonged psychological distress. Although national anxiety levels show post-pandemic recovery, these compounding factors may sustain elevated risks in minority adolescents, rendering them a sentinel population for monitoring long-term COVID-19 mental health sequelae.

Adolescent anxiety levels are influenced by various factors. Gender is a significant factor; a study on adolescent depression and anxiety symptoms found that girls reported more anxiety and depressive symptoms than boys (6). Left-behind children are another high-risk group. Previous research has shown that left-behind children score higher on anxiety scales compared to non-left-behind children (7), and during the 2019 COVID-19 lockdown, the prevalence of social anxiety and depression among left-behind children was significantly higher than after the lockdown (8). Another important factor is individual self-efficacy, or confidence in one’s abilities. Wang (9) found that general self-efficacy is negatively correlated with depression, state anxiety, trait anxiety, and test anxiety using the General Self-Efficacy Scale.

Based on existing research, this study proposes the following hypotheses: First, female high school students in the Tujia and Miao Autonomous Prefecture of Western Hunan will have higher anxiety levels than male students; second, left-behind high school students will have higher anxiety levels than non-left-behind students; and third, students with higher self-efficacy will have lower anxiety levels. To test these hypotheses, this study employs a cross-sectional survey design, collecting data through questionnaires administered to high school students in a school in the Tujia and Miao Autonomous Prefecture of Western Hunan. The completion of this study will help us understand the anxiety status of adolescents in this special region after the pandemic and provide scientific evidence for the development of targeted mental health interventions.

## 2 Methods

### 2.1 Participants

A convenience sampling method was used to select 426 students from the first and third grades of a minority high school in the Tujia and Miao Autonomous Prefecture of Western Hunan. Paper questionnaires were distributed to the students in February 2023, organized by class. To minimize potential confounding effects from event-specific stressors (e.g., examinations, grade announcements), this study strategically positioned its data collection during the second week of the academic semester. All participants signed informed consent forms, and the study was approved by the Ethics Committee of the School of Psychology at Shandong Normal University. After excluding incomplete and invalid questionnaires, 359 valid responses remained, yielding an effective response rate of 84.27%. Specific information is provided in Table 1.

**Table 1:** Demographic information of participants.

Variable	Frequency (n)	Percentage (%)	Variable	Frequency (n)	Percentage (%)
Gender			Mother’s educational level		
Male	189	52.6	Unknown	6	1.7
Female	170	47.4	No formal education	6	1.7
Grade			Primary school	64	17.8
First year	75	23.7	Junior high school	86	24.0
Third year	284	76.3	Senior high school or vocational school	121	33.7
Father’s educational level			Associate degree	34	9.5
Primary school	41	11.84	Bachelor’s degree or above	42	11.7
Junior high school	86	12.324	Left-behind		
Senior high school or vocational school	108	30.41	Yes	102	28.4
Associate degree	35	8.19.7	No	257	71.6
Bachelor’s degree or above	89	24.28			

### 2.2 Measurement instruments

The survey content in this study covers multiple aspects, including anxiety status, parental migration for work, parental educational level, and general self-efficacy. Self-efficacy was measured using the Chinese version of the General Self-Efficacy Scale revised by Wang (9). This scale consists of 10 items and uses a 4-point Likert scale (1 = Not at all true, 2 = Somewhat true, 3 = Mostly true, 4 = Exactly true). Higher scores indicate higher levels of self-efficacy. Anxiety status was assessed using the Generalized Anxiety Disorder 7-item scale (GAD-7) developed by Spitzer et al. (10) based on the diagnostic criteria of the Diagnostic and Statistical Manual of Mental Disorders, Fourth Edition (DSM-IV). Both domestic and international studies have shown that the GAD-7 has high reliability and validity for screening anxiety symptoms (10–13). The GAD-7 consists of 7 items, each rated on a 4-point scale (1 = Not at all, 2 = Several days, 3 = More than half the days, 4 = Nearly every day). Total scores range from 7 to 21, with scores of 7–10 indicating no or clinically insignificant anxiety, 11–15 indicating mild anxiety, 16–20 indicating moderate anxiety, and ≥21 indicating severe anxiety. To delineate the relationship between anxiety states and COVID-19 pandemic, we administered a pandemic impact severity scale (0–100; higher scores indicating greater perceived impact) to all participants, quantifying their self-reported psychosocial burden attributable to the ongoing pandemic.

### 2.3 Data analysis

Data analysis was conducted using IBM SPSS Statistics (version 21.0; IBM, Armonk, NY, USA). Independent samples t-tests were used to analyze differences in anxiety scores between different genders and between left-behind and non-left-behind adolescents. Correlation analysis and linear regression were employed to explore the relationships between various factors and anxiety levels. Correlation analyses were also conducted between anxiety levels and self-rated pandemic impact severity scores.

## 3 Results

### 3.1 Anxiety status

The results show that the prevalence of anxiety symptoms among the participants was 60.4%, with 16.4% experiencing moderate anxiety and 5.3% experiencing severe anxiety (Table 2).

**Table 2:** Prevalence of anxiety symptoms.

Anxiety level	Frequency (n)	Percentage (%)
No anxiety	142	39.6
Mild anxiety	139	38.7
Moderate anxiety	59	16.4
Severe anxiety	19	5.3

### 3.2 Differences in anxiety levels between genders

An independent samples t-test was conducted to examine the differences in anxiety levels between male and female high school students. The results showed that female students had significantly higher anxiety levels than male students (see Table 3).

**Table 3:** Differences in anxiety levels between the male and female.

	*M*	SD	*t*	*p*
Male	12.72	4.45		
Female	13.92	5.04	−2.38	0.02

### 3.3 Differences in anxiety levels between left-behind and non-left-behind adolescents

In this study, children or adolescents whose parents (one or both) work away from home for more than 4 months per year were classified as left-behind children or adolescents. An independent samples t-test was conducted to examine the differences in anxiety levels between left-behind and non-left-behind adolescents. The results showed that left-behind adolescents had significantly higher anxiety levels than non-left-behind adolescents (see Table 4).

**Table 4:** Differences in anxiety levels between the left-behind and non-left-behind.

	*M*	SD	*t*	*p*
Left-behind	14.31	4.82		
Non-left-behind	12.88	4.70	−2.58	0.01

### 3.4 Correlation analysis

To investigate the relationships between different factors and anxiety levels in adolescents, Pearson correlation analysis was conducted to examine the correlations between demographic variables and self-efficacy with anxiety levels. The results showed significant correlations between gender, being a left-behind child, and self-efficacy with anxiety levels. Specifically, self-efficacy was negatively correlated with anxiety levels (*r* = −0.20, *p* < 0.01) (see Table 5).

**Table 5:** Correlation analysis of various factors with the anxiety level.

Variable	*r*	*p*
Gender	0.13	0.02
Left-behind or not	0.14	0.01
Father’s educational level	−0.003	0.96
Mother’s educational level	−0.04	0.41
Self-efficacy	−0.20	0.00
Pandemic impact severity score	0.18	0.00

To delineate the relationship between anxiety states and COVID-19 pandemic, Pearson correlation analysis was conducted to examine the correlations between anxiety levels and self-rated pandemic impact severity scores. The significant correlation was also found (*r* = 0.18, *p* < 0.001) (see Table 5).

### 3.5 Linear regression analysis

Multiple linear regression was used to predict anxiety levels among high school students in the Tujia and Miao Autonomous Prefecture of Western Hunan, with gender, left-behind status, and self-efficacy as predictor variables. The results showed that gender, left-behind status, and self-efficacy collectively predict anxiety levels (*F* = 8.18, *p* < 0.01, *R*^2^ = 0.07) (see Table 6).

**Table 6:** Multiple linear regression analysis of predictors of anxiety level.

	*B*	*SE*	Beta	*t*	Sig
Constant	13.82	1.53		9.03	0.00
Gender	1.00	0.50	0.10	2.01	0.05
Left-behind or not	1.17	0.55	0.11	2.12	0.03
Self-efficacy	−0.15	0.04	−0.18	−3.44	0.00

## 4 Discussion

This study investigated the anxiety status and its influencing factors among high school students in the Tujia and Miao Autonomous Prefecture of Western Hunan, a minority concentration area, 3 years after the onset of the COVID-19 pandemic. The results indicate that 21.7% of the students experienced moderate to severe anxiety. Female high school students had significantly higher anxiety levels than male students, and left-behind children exhibited significantly higher anxiety levels compared to non-left-behind children. Students with higher self-efficacy had lower anxiety levels, and students who perceived greater contemporaneous psychosocial impacts from the COVID-19 pandemic exhibited significantly elevated anxiety levels. Gender, being a left-behind child, and self-efficacy collectively predict individual anxiety status.

The study found that 3 years after the pandemic, 21.7% of high school students in the Tujia and Miao Autonomous Prefecture of Western Hunan still experienced moderate to severe anxiety, and students who perceived greater contemporaneous psychosocial impacts from the COVID-19 pandemic exhibited significantly elevated anxiety levels. These results highlight that the anxiety levels among adolescents in this area remain quite severe, and this severe situation is closely related to the COVID-19 pandemic. Among these students, female high school students showed significantly higher anxiety levels than their male counterparts. This finding aligns with previous research, which indicates that women are more likely to experience anxiety when facing stress (6). Additionally, the roles and expectations placed on women in both family and society may contribute to their psychological burden. In minority regions, this gender difference may be more pronounced, as female students face additional family and social responsibilities alongside academic pressures. Therefore, schools and families should provide more psychological support to female students to help them better manage stress and improve their mental health.

The study also revealed that left-behind children had significantly higher anxiety levels compared to non-left-behind children. Chen and Guo (14) noted that the absence of parental companionship due to parents working away from home leads to insufficient emotional support within the family, resulting in feelings of inferiority, anxiety, and marginalization among left-behind children. Other studies have shown that the anxiety levels of left-behind children are associated with lower quality of life, poorer family function, more siblings, physical abuse, gender (female), and ethnicity (minority) (15). In minority regions, many families must work away from home to earn a living, leading to a significant number of left-behind children. Therefore, the government and society should increase support for these children, providing more psychological counseling and educational resources to help them build confidence and enhance their psychological resilience.

Our study found that students with higher self-efficacy had lower anxiety levels. Individuals with higher self-efficacy tend to adopt more effective self-regulation strategies when facing challenges and stress. They are better able to monitor, regulate, and control their cognition, motivation, and behavior, thereby reducing anxiety (16). Emotionally, individuals with high self-efficacy can maintain a positive emotional state when encountering difficulties, reducing the impact of negative emotions such as anxiety and depression (17). In minority regions, enhancing students’ self-efficacy is particularly important. Schools can achieve this by organizing various activities and courses to foster students’ confidence and coping skills, helping them better handle academic and life challenges. Parents and teachers should also encourage students to participate in social practice activities to boost their self-efficacy.

The results also show that gender, being a left-behind child, and self-efficacy can collectively predict individual anxiety status. Gender differences and being a left-behind child reflect the influence of external environmental factors on students’ psychological well-being, while self-efficacy reflects internal psychological resources. Considering these factors comprehensively can provide a more thorough understanding of students’ anxiety status and inform the development of more effective intervention measures. For example, schools and families can offer more psychological support and resources to female students and left-behind children, while also working to enhance students’ self-efficacy to reduce their anxiety levels and improve their mental health.

Despite the valuable insights provided by this study, there are some limitations. First, the study used a cross-sectional design, which cannot establish causality and can only reveal associations between variables. Future research could employ a longitudinal design to explore the long-term trends and effects of these factors on anxiety status. Second, the study did not consider potential influencing factors such as family economic status and family history of anxiety disorders. Future research should further investigate the role of these factors. Third, this study lies in its exclusive reliance on self-reported paper-based anxiety measures rather than clinician-administered evaluations. While we employed psychometrically validated instruments such as the Generalized Anxiety Disorder 7-item (GAD-7) scale, these screening tools cannot supplant comprehensive diagnostic assessments conducted by licensed mental health professionals. Future investigations could strengthen methodological rigor through multi-modal approaches incorporating structured clinical interviews alongside psychometric inventories to corroborate findings.

In conclusion, 3 years after the pandemic, the anxiety status of high school students in the Tujia and Miao Autonomous Prefecture of Western Hunan remains severe, with females, left-behind children, and individuals with low self-efficacy being at higher risk. These findings provide a scientific basis for developing targeted mental health interventions. It is recommended that schools and communities increase their focus on these high-risk groups, providing psychological support and counseling to help them better cope with stress and promote their mental health development. In addition, schools can cultivate students’ confidence and coping skills through structured extracurricular programs and resilience-focused curricula, equipping them to navigate academic and psychosocial challenges. Concurrently, parents and educators should facilitate skill-transfer opportunities by mentoring students’ participation in community-engaged service-learning projects, thereby enhancing self-efficacy through mastery experiences.

## Data Availability

The raw data supporting the conclusions of this article will be made available by the authors, without undue reservation.
